# The Taste of Sustainability: Sensory Experience and Stated Preference Trade-Offs in Consumer Evaluation of Goat Cheese from Extensive Farming Systems

**DOI:** 10.3390/foods14183197

**Published:** 2025-09-13

**Authors:** Giuseppe Di Vita, Manal Hamam, Luigi Liotta, Vincenzo Lopreiato, Maria Lunetta, Federica Consentino, Daniela Spina

**Affiliations:** 1 Department of Agriculture, Food, and Environment, University of Catania, 95123 Catania, Italy; giuseppe.divita1@unict.it (G.D.V.); federica.consentino@outlook.it (F.C.); daniela.spina@unict.it (D.S.); 2 Research Centre for Agricultural Policies and Bioeconomy, CREA, 00187 Rome, Italy; 3 Department of Veterinary Sciences, University of Messina, 98168 Messina, Italy; luigi.liotta@unime.it (L.L.); vincenzo.lopreiato@unime.it (V.L.); maria.lunetta@studenti.unime.it (M.L.)

**Keywords:** sustainable cheese, mediating role, claims, consumer attitude, intention to buy, PLS-SEM, sensory experiment

## Abstract

This research investigates consumer behavior and intention to buy (ITB) for sustainable goat cheese made from milk sourced through extensive farming systems. By integrating sensory experiment with stated preference data on credence and search attributes—such as sustainability claims, labeling, and quality certifications—and analyzing them using Partial Least Squares Structural Equation Modeling (PLS-SEM), this research offers a comprehensive perspective on the drivers of consumer decision-making, bridging actual sensory perception with hypothetical market choices. The findings clarify the trade-offs consumers are willing to make between taste and sustainability. Notably, the results reveal that a compelling sensory experience can lead consumers to deprioritize sustainability indicators and labeling claims, indicating that when sensory satisfaction is high, informational cues exert less influence on purchase intentions. To deepen the analysis, this study also explores the mediating role of consumer attitude, demonstrating that attitudes significantly translate product perceptions—particularly sensory and extrinsic attributes—into buying intentions. This integrated approach contributes a novel methodological framework and offers both theoretical and practical insights for marketers and policymakers aiming to promote sustainable food choices.

## 1. Introduction

Consumer behavior in the agri-food sector is shaped by the interplay of sensory, extrinsic, and credence attributes [1]. In the dairy industry, intrinsic qualities such as aroma, appearance, taste, and texture remain crucial drivers of hedonic evaluation [2,3], while extrinsic and credence cues—such as labeling, certifications, and origin—add cognitive value by anchoring expectations and building trust [4,5]. As sustainability concerns rise, the origin of raw materials and farming systems has become increasingly relevant [6], with extensive systems perceived as more animal-friendly and environmentally sustainable, thus appealing to ethically conscious consumers [7]. Nonetheless, sensory satisfaction continues to dominate cheese acceptance, often outweighing informational claims [2,8].

Goat cheese provides an ideal case study, as it combines artisanal methods, strong sensory distinctiveness, and increasing consumer interest linked to health, tradition, and sustainability [9]. Global goat milk production reached 20.9 million tonnes in 2023, and the goat cheese market is valued at USD 8.21 billion in 2025, with Europe (particularly Italy, Spain, and the Netherlands) accounting for 38.42% of the market [10,11]. Consumer preferences for goat cheese are evolving, shaped by dietary shifts, sustainability awareness, and demand for premium products [12,13]. Sensory qualities such as texture, flavor balance, and appearance remain highly valued [14], though preferences vary regionally [15].

A rich body of research highlights that sensory attributes and sustainability cues shape food choices through distinct pathways: sensory traits influence hedonic responses, while extrinsic and credence claims (price, certification, packaging) affect cognitive evaluations [16,17]. Yet, studies rarely integrate these domains to assess how they interact in shaping purchase intentions.

This study addresses this gap by combining sensory experiments with stated preference data on credence and extrinsic attributes (e.g., sustainability claims, quality certifications, labeling). Specifically, it investigates how consumers trade-off sensory satisfaction with sustainability indicators and examines the mediating role of consumer attitudes in translating perceptions into purchase intentions. Goat farming under extensive systems provides an especially relevant context, as it supports biodiversity, rural livelihoods, and territorial development [18,19], but higher production costs require effective communication to capture consumer value [20,21].

This research further tests the expectation–disconfirmation effect, comparing label-driven expectations with post-tasting evaluations of goat cheese from extensive systems to assess how cues align—or conflict—with actual sensory experience. Using Partial Least Squares Structural Equation Modeling (PLS-SEM), this study evaluates:

(a) How consumers balance sensory attributes (taste, texture, aroma, appearance) with extrinsic/credence cues (labels, claims, certifications) in purchase intentions;
(b) The mediating role of consumer attitudes in this relationship.

By integrating sensory and credence perspectives, this study advances the understanding of consumer evaluation of sustainable dairy products and provides actionable insights for producers, marketers, and policymakers seeking to enhance the competitiveness of goat cheese while promoting sustainable food consumption.

## 2. Conceptual Background

### Research Hypotheses

The conceptual model is structured around the idea that consumer decision-making for goat cheese is shaped by a combination of intrinsic (implicit) sensory cues and extrinsic (explicit/credence) cues, which, together, influence attitudes and ultimately the intention to buy. In the marketing literature, intrinsic (implicit) factors—such as taste, aroma, texture, and appearance—refer to product characteristics directly experienced by the consumer. These sensory dimensions act as primary reference points in food evaluation, forming the hedonic basis of consumer satisfaction and repeat purchase behavior [10,22].

By contrast, extrinsic (explicit) factors—such as price, branding, geographical indications, certifications (e.g., PDO, organic, animal welfare), sustainability claims, and labeling—are not directly experienced but provide symbolic, informational, and ethical value. These signals act as external reference points that help consumers reduce uncertainty, build trust, and justify premium prices [18,19].

Consistent with the objectives of this study, and following recent research on consumer trends in the agri-food sector, this investigation is structured around five key areas:

(a) Sensory attributes

Sensory attributes are fundamental in shaping consumer preferences for cheese, influencing both initial selection and long-term purchasing behavior [23]. Research consistently demonstrates that taste, texture, aroma, and appearance are key determinants of consumer satisfaction and willingness to buy [2].

In the case of goat cheese, sensory characteristics play an even more decisive role. Its distinct flavor profile can attract certain consumers while deterring others, making taste and texture critical factors in purchase decisions [15]. Additionally, recent studies highlight a growing preference for small-scale and artisanal goat cheeses over mass-produced varieties. Indeed, consumers seek unique flavors and textures that reflect traditional craftsmanship [19]. Considering this, the following hypotheses are proposed:

**H1a:**  *Sensory characteristics have a direct and positive impact on consumers’ attitude in purchasing goat cheese.*

**H1b:**  *Sensory characteristics have a direct and positive impact on consumers’ intention to buy goat cheese.*

(b) Extrinsic characteristics

Extrinsic characteristics—such as price, promotions, brand reputation, and packaging sustainability—play a crucial role in shaping consumer preferences for goat cheese and are key purchasing determinants.

Several studies highlight the influence of price on consumers’ perceptions of quality and value. In choosing goat cheese and related products, consumers often associate higher prices with premium quality as artisanal production, or organic certification, while budget-conscious buyers may prioritize affordability over sensory attributes [13,24].

Discounts, bundle offers, and in-store tastings can significantly impact purchasing decisions [25]. Consequently, promotional strategies help introduce consumers to new products, reduce perceived risk, and increase trial rates [26], particularly for niche markets like goat cheese.

Even trust in a brand can enhance consumer willingness to pay a premium. Brands with strong reputations for authenticity, tradition, and sustainability tend to attract loyal customers who prioritize quality and ethical sourcing [27].

Finally, eco-friendly packaging can influence purchasing choices. In fact, food consumers are increasingly drawn to brands that use recyclable or biodegradable materials [28], aligning with their environmental values and reducing guilt associated also with consumption of dairy products [5,29]. Considering this, the following hypothesis is proposed:

**H2a:**  *Extrinsic characteristics have a direct and positive impact on consumers’ attitudes about interest in purchasing goat cheese.*

**H2a:**  *Extrinsic characteristics have a direct and positive impact on consumers’ intention to buy goat cheese.*

(c) Importance of Geographical indication and production method claims

Geographical origin plays a crucial role in shaping consumer perceptions of authenticity, quality, and tradition in food products [30]. Consumers are increasingly interested in the geographical origin and production methods of the milk used in cheesemaking. Goat cheeses labeled with details about extensive (pasture-based) farming practices tend to be more appealing, as they are associated with higher animal welfare standards and environmental sustainability [31]. Additionally, certifications such as Protected Designation of Origin (PDO) and mountain labels enhance perceptions of authenticity and quality, influencing consumers’ willingness to pay for dairy products [13].

Consumer awareness of animal welfare has grown significantly, with increasing demand for products that prioritize ethical treatment of animals [32]. Research indicates that goat cheese carrying animal welfare certifications is often perceived as more desirable [33]. Similarly, sustainability considerations play an essential role in purchasing decisions, as eco-friendly production practices resonate with environmentally conscious consumers [28]. Moreover, organic production methods positively influence purchase intent, as consumers associate them with superior animal welfare and environmental sustainability [34,35]. Considering this, the following hypotheses are proposed:

**H3a:**  *Quality claims (certified production methods—such as organic certification, PDO designation, mountain labels, and animal welfare certifications) have a direct and positive impact on consumers’ attitudes about interest in purchasing goat cheese.*

**H3b:**  *Quality claims (certified production methods such as organic certification, PDO designation, mountain labels, and animal welfare certifications) have a direct and positive impact on consumers’ intention to buy goat cheese.*

(d) Product origin and label information

Milk provenance, whether locally sourced or derived from specific breeds, is particularly important in consumer preferences. Studies suggest that locally sourced milk is often favored due to its perceived freshness and the support it provides to local farmers [36].

Label information is one of the extrinsic cues that influences consumer trust and purchasing decisions in the cheese market. Indeed, labels provide essential details about a product’s origin, nutritional content, and production methods, significantly influencing consumer perceptions and behavior [5].

Additionally, nutritional labeling, which highlights fat content, protein levels, and additives, helps consumers assess the health benefits of dairy products. This transparency is especially valued by health-conscious consumers [37].

Beyond factual product details, tasting tips featured on packaging or promotional materials further guide consumer choices. Research suggests that descriptive sensory labeling can elevate expectations and positively influence perceived flavor quality, making it a powerful tool in shaping purchasing behavior [38]. Considering this, the following hypotheses are proposed:

**H4a:**  *Label information (milk provenance, product origin, label information, and tasting tips) has a direct and positive impact on consumers’ attitudes about interest in purchasing goat cheese.*

**H4b:**  *Label information (milk provenance, product origin, label information, and tasting tips) has a direct and positive impact on consumers’ intention to buy goat cheese.*

(e) Attitude and Mediation analysis

Attitude reflects an individual’s predisposition, whether favorable or unfavorable, toward a specific activity or behavior [39]. Research has identified attitude as a key predictor in mediating the relationship between various influencing factors [40,41]. Moreover, a direct link between attitude and the inclination to consume and purchase food has been revealed [42].

Thus, understanding attitudes toward food behavior is crucial for examining the determinants of purchase intention. Based on this, the following hypotheses are proposed:

**H5a:**  *Attitude has a direct and positive effect on consumers’ intention to buy for goat cheese.*

Although numerous studies document that both product attributes (sensory, extrinsic, and credence cues) and attitudinal evaluations influence buying decisions [16], the mechanisms by which attribute perceptions are transformed into intentions remain underexplored. Mediation analysis enables us to test whether—and to what extent—attitude explains the effect of product attributes on purchase intention [43]. In fact, mediation analysis allows researchers to understand the causal mechanism underlying the relationship between two variables [44,45] and provides a comprehensive understanding of consumer decision-making, guiding focused measures to encourage the adoption of choices [45]. Beyond the direct effect that one construct can exert on another, independent constructs can also have an indirect effect [46]. Since PLS-SEM models are often applied to complex frameworks with multiple relationships, a multi-path mediation effect or multiple mediating constructs within a single path may occur [47]. When testing mediation effects, it may be found that the indirect effect is not significant, indicating a situation of no mediation [46]. However, if significance is confirmed, mediation can be classified as full mediation (the direct effect is not significant), competitive partial mediation (direct and indirect effects have opposite directions), or complementary partial mediation (indirect and direct effects point in the same direction) [47].

**H5b:**  *Attitude mediates the effect of sensory characteristics on consumers’ intention to buy for goat cheese.*

**H5c:**  *Attitude mediates the effect of extrinsic characteristics on consumers’ intention to buy for goat cheese.*

**H5d:**  *Attitude mediates the effect of quality claims on consumers’ intention to buy for goat cheese.*

**H5e:**  *Attitude mediates the effect of label information on consumers’ intention to buy for goat cheese.*

**Figure 1:**
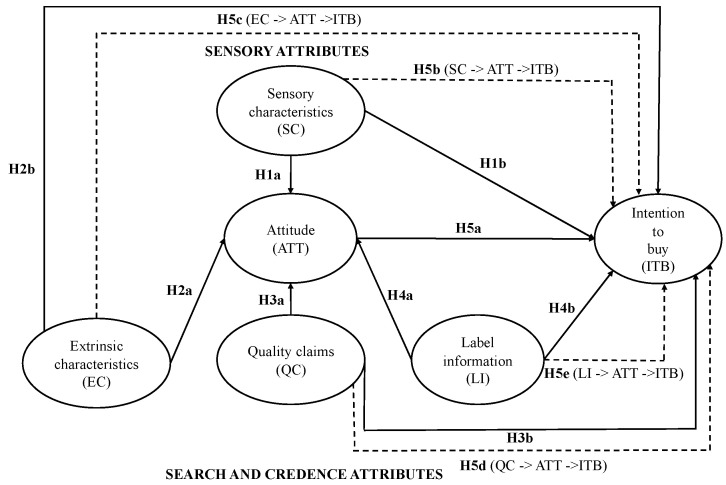
Research hypotheses graph.

Figure 1 illustrates the comprehensive model representing this conceptualization.

## 3. Materials and Methods

### 3.1. Data Collection and Survey Design

The survey was conducted in Floresta, a small village in the Nebrodi Park (province of Messina), which is the highest municipality in Sicily (Italy), at approximately 1300 m above sea level. Data collection took place every Sunday throughout October 2024 during Ottobrando, the most prominent regional food and wine event, at the institutional booth of the iSAFE GRAZE Project. The event attracts around 10,000 visitors each Sunday from the regions of Sicily and Calabria, driven by a strong interest in locally produced, sustainably sourced products that respect animal welfare and the environment.

A total of 100 consumers were randomly recruited among the event visitors. Eligibility was confirmed to ensure participants did not have an aversion to goat cheese.

The sample size met the requirements established by Resurreccion [48]. A one-sample t-test was used to determine the statistical power (99.86%) associated with the reference sample, based on the expected effect and the standard deviation.

The participants were 59.00% male and 41.00% female.

The participants were aged between 18 and 80 years, with an average of approximately 43 years.

The survey design was structured in two main phases: a sensory test and an assessment of stated preferences. By integrating both sensory evaluation and stated preference analysis, this study provided a comprehensive understanding of consumer behavior, bridging actual sensory perception with hypothetical market choices. The sociodemographic characteristics of the sample are presented in Table 1.

### 3.2. Sensory Experiment

In this phase, the participants were invited to evaluate the cheese sample under controlled conditions. The evaluation focused on key sensory attributes, including appearance, texture, aroma, and taste. The respondents rated each characteristic using a 5-point Likert scale, ranging from “Strongly disagree” (1) to “Strongly agree” (5). This step aimed to capture the perceived quality and organoleptic appeal of the products.

The cheese used in the test was crafted from raw milk obtained from indigenous Rossa Mediterranean goats reared in an extensive system. The cheesemaking process included the following steps: curd formation, cooking in whey (80 °C for 1.5 h), drying (2 days), salting with dry salt (once per week for 4 weeks), and aging (60 days). After maturation, the 1 kg cheese wheels were vacuum-packed, labeled with the project-specific branding, and stored at +4 °C until testing. Prior to the sensory sessions, the packaging was removed, and the cheese was allowed to reach room temperature to ensure optimal sensory perception.

Each participant was invited to taste an artisanal goat cheese produced under extensive farming conditions, with pasture-based feeding supplemented minimally with simple feedstuffs and agro-industrial by-products (e.g., grape pomace, citrus pulp, and brewing by-products). The participants were asked to taste the sample and complete a questionnaire to score their sensory impressions. The questionnaire was accessed via a QR code and completed digitally through Google Forms.

The participants were anonymized using unique identification numbers. Before the test began, a brief explanation of the sensory attributes and the intensity scale was provided to ensure all participants could confidently engage in the evaluation, which lasted approximately 30 min per session.

The primary objective was to evaluate the influence of sensory attributes on consumers’ intention to buy.

Ethical approval for the involvement of human subjects in this study was granted by the University Research Ethics Committee of the Department of Veterinary Sciences (University of Messina), reference number May 2024, dated 23 May 2024. The participants gave informed consent via the statement “I am aware that my responses are confidential, and I agree to participate in this survey”, for which an affirmative reply was required to enter the survey. They were able to withdraw from the survey at any time without giving a reason.

### 3.3. Stated Preference Survey

After the sensory test, the participants completed a structured questionnaire designed to assess their stated preferences and purchase intentions.

The questionnaire consisted of 6 sections and contained 26 items addressing sensory characteristics (aroma, appearance, taste, and texture), extrinsic characteristics (price, promotions, brand reputation, and recyclable packaging), quality claims (organic certification, PDO certification, mountain product certification, and indication and/or certification animal welfare), label information (place of provenance of the milk, place of processing of the product, nutritional information on the label, and tasting tips), attitude, intention to buy, and sociodemographic characteristics (gender, age, education, and income).

The evaluation of sensory characteristics, extrinsic attributes, claims, and label information was conducted using a 5-point Likert scale, where 1 represented “Strongly disagree” and 5 denoted “Strongly agree”.

To assess consumer attitude, the participants were asked: “I may be interested in purchasing this cheese”. The responses were recorded on a 1-to-5 Likert scale, with 1 indicating “Strongly disagree” and 5 “Strongly agree” (Table 2).

Norman [49] suggests that Likert scales produce reliable findings in parametric analyses, even under conditions of uneven variance, small sample sizes, and nonnormal distributions.

To measure intention to buy (ITB) at a premium price, the respondents answered: “I intend to buy goat cheese”. The response options were: 1 = low; 2 = medium; 3 = high.

### 3.4. Data Analysis Based on Partial Least Squares Structural Equation Modeling (PLS-SEM)

The empirical analysis sought to examine the links among the following constructs: “sensory characteristics”, “extrinsic characteristics”, “quality claims”, “label information”, “attitude”, and “intention to buy”.

The Partial Least Squares Structural Equation Modeling (PLS-SEM) method was adopted as it is considered particularly suitable for predictive analysis purposes.

PLS-SEM is a multivariate analytical method within the structural equation modeling framework and is extensively employed in both experimental and observational contexts to investigate food purchasing behavior and consumer preferences [50].

In comparison to other methodologies, it offers two advantages: the incorporation of latent variables inside the model and the capability to estimate numerous dependence relationships [51].

The PLS-SEM methodology is favored over CB-SEM (Covariance-Based Structural Equation Modeling) due to its less rigorous assumptions on normality and error distributions and its reduced sensitivity to sample size [52]. Nonetheless, several restrictions exist regarding the increased probability of neglecting authentic links and the sensitivity to the comparative magnitude of descriptive variables [53].

This method has been effectively utilized in prior studies to evaluate the Theory of Planned Behavior (TPB) [54] and consumer perceptions on food selections [55].

PLS-SEM has been frequently used in the literature for sensory analysis studies [56,57], precisely because it is well suited to contexts characterized by small sample sizes. This approach is particularly appropriate for sensory evaluations, where the number of participants is often limited [58]. In fact, PLS-SEM allows for the estimation of models even with small samples, maintaining good predictive power and a certain degree of statistical robustness, making it a particularly useful technique in this field of research [57].

The PLS-SEM model comprises estimations derived from two components: an external measurement model and an internal structural model.

The external model analyzes the relationships between latent variables and their indicators [59,60].

This study posits that the relationship is reflexive, meaning that indicators represent a manifestation of the underlying notion [61].

The internal model, conversely, illustrates the links (paths) among constructs (latent variables) regarded as components rather than common factors in PLS-SEM. Path coefficient values were utilized to assess the structural model [62].

These interactions may be direct or indirect based on the presence or absence of a mediating variable.

Following the evaluation of the indicator’s dependability (factor loadings > 0.5) [63], indices like Cronbach’s alpha (CA), Dillon–Goldstein (DG) rho coefficient, and rho coefficient A > 0.6 were utilized to determine internal consistency in exploratory study A [64].

The reliability of the questionnaire was assessed by evaluating the Cronbach’s alpha coefficient. Alpha values between 0.6 and 0.7, as well as 0.8, are deemed acceptable, although a value over 0.8 signifies outstanding reliability [65].

The discriminant and convergent validity of the measuring model were subsequently assessed.

Convergent validity is confirmed when the average variance extracted (AVE) of the construct is 0.5 or higher [66]. Discriminant validity is determined by the Fornell–Larcker [67] criterion, which entails comparing the square root of the Average Variance Extracted (AVE) with the correlation among latent constructs [62] (Table A1). It indicates the degree to which a specific notion varies from others based on empirical criteria [68].

An evaluation of collinearity amongst constructs was performed to determine the degree of common method bias by analyzing variance inflation factors [69].

The variance inflation factor (VIF) values for all variables were below 3, signifying no substantial collinearity among constructs [55] (Table A2).

Statistical analyses were conducted using Stata 18 (StataCorp LP, College Station, TX, USA).

## 4. Results

### 4.1. PLS-SEM Results

#### 4.1.1. The Measurement Model Output

The outcomes derived from the measurement model are summarized in Table 3. To evaluate the model’s validity [62], the researchers initially analyzed the relationships between the latent components and their corresponding items, emphasizing the reliability of the indicators. The standardized external factor loading for all 26 items was deemed satisfactory as it surpassed the threshold value of 0.45 (*p* < 0.001) [68].

All constructs analyzed had Cronbach’s alpha and rho A coefficients greater than 0.7, and the Dillon–Goldstein (DG) rho coefficient surpassed 0.8. This signifies adequate levels of internal consistency [70]. Constructs with values exceeding 0.5 are deemed to demonstrate convergent validity [71].

The constructs demonstrated good discriminant validity, surpassing the requisite threshold of 0.5 [72]. The square root of the Average Variance Extracted (AVE) values exceeded the correlations with other constructs, following Fornell–Larcker’s [67] criterion (Table A1).

**Table 3:** Measurement model results.

	Sensory Characteristics	Extrinsic Characteristics	Quality Claims	Label Information	Attitude	Intention to Buy
ARO.1	0.786					
ARO.2	0.838					
ARO.3	0.621					
APP.1	0.807					
APP.2	0.775					
APP.3	0.809					
TAS.1	0.781					
TAS.2	0.854					
TAS.3	0.470					
TEX.1	0.557					
TEX.2	0.677					
TEX.3	0.707					
PRICE		0.671				
PROM.		0.782				
BRAND.REP		0.825				
RECK.PACK		0.710				
ORGANIC.CERT			0.867			
PDO.CERT			0.891			
MOUNT.CERT			0.846			
ANIM.CERT			0.774			
MILK.PROV				0.709		
PROD.ORIG				0.798		
NUTR.INFO				0.944		
TASTING.TIPS				0.880		
ATT					1.000	
INTENTION TO BUY						1.000
Cronbach	0.918	0.757	0.868	0.864		
DG	0.931	0.836	0.909	0.903		
rho_A	0.932	0.761	0.887	0.967		

#### 4.1.2. The Structural Model Output

**Figure 2:**
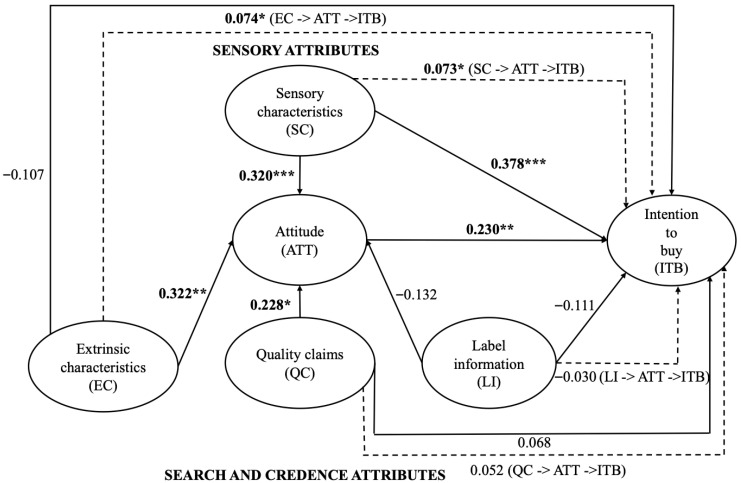
Graph of structural model results. Note: *, **, and *** denote significance at 10%, 5%, and 1% levels, respectively.

The outcomes for the direct and indirect effects of the structural model are displayed in Table 4 and Figure 2. Latent constructions are depicted as ovals, and the presumed links between constructs are represented by arrows.

Path coefficients indicate the strength and direction of the direct relationship between two constructs, whereas R^2^ evaluates the adequacy of the structural model [72]. An R^2^ value of 0.31 signifies that the suggested model possesses a good capacity to predict the intention to buy for goat cheese [68].

The assessment of the structural model’s fit indicated that each route coefficient conformed to the anticipated trajectory.

The results do not confirm all hypotheses regarding associations between constructs, among them: direct relationships between “extrinsic characteristics” and “intention to buy”, “quality claims” and “intention to buy”, “label information” and “attitude”, “label information” and “intention to buy”; and indirect relationships between “quality claims” and “intention to buy”, and “label information” and “intention to buy”.

Substantial coefficients were found for the remaining hypotheses, indicating a good fit of the proposed path model, as evidenced by a *p*-value of less than 0.10.

The findings corroborated the H1a hypothesis that “sensory characteristics” considerably affect customers’ “attitude” towards purchasing goat cheese.

The findings for “credence attributes” corroborated hypotheses H2a and H3a, indicating that “extrinsic characteristics” is the most important predictor (*p* > 0.001), followed by “quality claims” (H3a) (*p* < 0.10). The findings, however, did not corroborate the H4a theory that “label information” has no meaningful influence on “attitude” (*p* > 0.10).

The findings further corroborated hypothesis H5a, indicating that “attitude” has a direct influence on “intention to buy” (*p* < 0.05).

The results indicated that only hypotheses H5b and H5c were supported for indirect links with “intention to buy”, where “sensory characteristics” and “extrinsic characteristics”, respectively, affect “intention to buy” via “attitude”. The findings did not support hypotheses H5d and H5e (*p* > 0.10), showing that “attitude” does not facilitate the transmission of information between “quality claims” and “intention to buy”, as well as “label information” and “intention to buy”.

The outcomes of Sobel’s test further validated the mediating effect of attitude for H5b and H5c. Specifically, “attitude” mediates the relationship between “sensory characteristics” and “intention to buy” (β = 0.073; z = 1.767; SE = 0.042; *p* = 0.077), as well as between “extrinsic characteristics” and “intention to buy” (β = 0.074; z = 1.728; SE = 0.043; *p* = 0.084).

Conversely, “attitude” did not enhance the correlation between “quality claims” and “intention to buy” (β = 0.052; z = 1.401; SE = 0.037; *p* = 0.161) (H5d), nor between “label information” and “intention to buy” (H5e) (β = −0.030; z = −0.990; SE = 0.031; *p* = 0.322).

## 5. Discussion

The PLS-SEM analysis reveals that sensory characteristics, extrinsic attributes, and quality claims (e.g., certifications and animal welfare) all significantly influence consumer attitudes toward artisanal goat cheese from extensive farming systems. This finding directly addresses the first research question (RQ1), which explores the extent to which consumers are willing to trade-off sensory attributes—such as taste, aroma, texture, and appearance—against search and credence cues, including price, packaging, quality labels, and origin information, when forming purchase intentions. Among the variables examined, sensory appeal emerged as the strongest predictor of purchase intention. This emphasizes the trade-off central to RQ1: when sensory satisfaction is strong, ethical and informational cues become less influential in shaping purchase intentions.

These findings corroborate our hypothesis and align with prior research emphasizing the critical role of sensory experiences in shaping food preferences [2]. They reaffirm that sensory appeal remains a dominant driver in consumer decision-making, largely due to its ability to evoke emotional responses and create a more engaging consumption experience [73].

The in-person tasting further reinforced the importance of sensory characteristics, demonstrating how direct interaction with the product can significantly shape consumer attitudes, particularly for niche items like artisanal cheeses [23]. This experiential component aligns with the literature suggesting that hands-on engagement enhances perceived authenticity and strengthens the consumer-product connection [74].

Previous research showed that sensory attributes are primary drivers of hedonic evaluations, whereas extrinsic and credence cues operate more cognitively, anchoring expectations and trust [16]. Our results support this dual-process view: sensory appeal remains the strongest predictor of purchase intention, even when consumers are exposed to rich sustainability messaging. Similar patterns have been observed across different food categories, with taste and texture consistently dominating preferences over extrinsic or credence cues [74]. For example, in research on Cheddar cheese raised under different feeding regimes, taste was the main driver of liking regardless of farming inputs [75].

Sustainability claims can interact with sensory perceptions through the expectation–disconfirmation framework [74]. Consumers often anticipate superior taste from “organic” products but may experience disconfirmation when the actual sensory experience falls short [75]. Our analysis reveals such effects in goat cheese from extensive farming systems, highlighting the potential mismatch between sustainability cues and sensory reality.

From a methodological standpoint, our modeling approach is grounded in a broader body of research that employs PLS-SEM to capture the joint influence of sensory and extrinsic attributes. For example, Tenenhaus et al. [76] applied PLS regression to link consumers’ liking of products with both their physico-chemical and sensory characteristics. More recently, da Veiga et al. [56] used PLS-PM to show that sensory appeal is the main factor driving interest in low-sugar products. Similarly, Cela et al. [77] predicted consumers’ attitudes and purchase intentions for beers brewed with agro-industrial by-products, demonstrating how sensory and ethical factors differently affect choices. Hamam et al. [47] applied PLS-SEM to study the mediating role of attitudes toward innovative products, such as pork from pigs fed with insect flour, highlighting the method’s usefulness in examining how perceptions and attitudes influence purchase intentions.

Integrating implicit sensory tests—such as non-verbal reaction-time tasks or physiological measures—would allow researchers to capture unconscious gustatory responses [78,79,80,81] and reveal whether “sustainable” labels truly enhance hedonic perception or merely inflate self-reports. Such techniques can uncover hidden attitudes that drive actual consumption [82].

Finally, the role of sensory characteristics in shaping consumer preferences is well documented, with flavor identified as the most decisive factor influencing liking and purchase intentions [83], though preferences vary by cheese type [84]. Aroma is also critical, with consumers favoring distinctive, intense profiles over standardized options [85]. These results substantiate existing evidence that aromatic complexity enhances the sensory appeal of artisanal cheeses [86].

Texture, a marker of artisanal production, contributes to perceived authenticity and has been emphasized in studies on Serrano and Canastra cheeses [87]. Appearance also shapes quality perceptions, through visual cues like color and crust. These findings align with evidence that sensory profiles in artisanal cheeses reflect both craftsmanship and raw material quality [3], while environmental factors such as pasture feeding enhance flavor intensity [88].

In addition to sensory characteristics, extrinsic attributes and product claims emerged as significant contributors to consumer attitudes, reinforcing the multifaceted nature of decision-making processes. These results corroborate earlier research highlighting the importance of promotional strategies, brand reputation, geographic origin, and declared information about animal welfare in shaping consumer perceptions [6,89].

The findings confirm that quality labels are vital for fostering consumer trust and product appeal [5]. Ethical and environmental claims increasingly influence consumer attitudes, as seen in the case of goat dairy products, which reflect growing concern for animal welfare and sustainability [90].

Our outcomes also evidence that label information has a weaker impact on consumer attitudes and does not significantly mediate the willingness to pay a premium price. This outcome is consistent with prior studies indicating that direct sensory experiences often exert a stronger influence on consumer perceptions than extrinsic cues, particularly for artisanal products [91].

The outcomes of this study strongly evidence the mediating role of consumer attitude in the relationship between sensory attributes and search and credence attributes (extrinsic, claims, and labeling attributes) with respect to the intention to buy (ITB) (RQ2).

Aligned with research on consumer psychology [78], this study confirms attitude functions as a key psychological mediator between product evaluations and purchase intentions. This mediation is strongest for sensory characteristics and extrinsic attributes, supporting the view that initial perceptions shape attitudes, which, in turn, drive buying behavior.

A weaker but still significant mediation effect is observed for product claims, suggesting that ethical and environmental claims impact purchase intention primarily when they foster a favorable attitude. This is in agreement with evidence that consumers are more likely to act on value-aligned claims when the product is also seen as credible and appealing [92].

However, label information did not show a significant direct or mediated effect on willingness to pay a premium price, indicating that consumers may not rely on labeling as a primary driver of their purchasing decisions in this category. As noted, the impact of labeling tends to be contingent upon consumers’ prior knowledge and personal involvement in food choices [93,94].

Contrary to initial expectations, demographic variables such as education, gender, and income did not significantly influence willingness to pay a premium price. This lack of significance suggests that interest in artisanal goat cheese is cross-cutting and not confined to specific demographic groups. This is likely due to the tasting experience reducing pre-existing differences, leading to more consistent appreciation across diverse consumer segments. It underscores the potential of direct sensory engagement to broaden demographic boundaries and expand the market appeal of artisanal goat cheese.

## 6. Conclusions

This study provides an innovative contribution to the existing literature by simultaneously examining the effects of direct product tasting on preferences for search and credence attributes and the mediating role of consumer attitudes on the intention to buy artisanal goat cheese from extensive farming systems. This dual approach, applied for the first time to cheese, highlights the value of integrating sensory experience into consumer research.

The findings reveal that sensory characteristics significantly influence consumer attitudes, which positively affect purchase intent. Direct tasting enhanced subjective perceptions, strengthening attitudes and consequently purchase intentions. The mediating role of attitude suggests that, without favorable sensory experiences, the impact on purchase would be weaker. These results support previous research showing how tasting opportunities help build trust and appreciation for sustainable food products.

Sensory appeal emerged as the most influential factor, with intrinsic attributes such as appearance, taste, and flavor central in shaping consumer preferences. Extrinsic attributes, including packaging, geographic origin, and sustainability claims, also shape attitudes, but to a lesser degree than sensory experiences. Claims emphasizing artisanal nature and sustainable origin positively influenced attitudes and willingness to buy, though their mediated effect was weaker than that of sensory attributes. Label information had only a marginal influence, as consumers prioritized direct sensory perceptions over external details, consistent with prior studies on artisanal products.

Attitude emerged as the key variable linking product attributes to purchase intentions, highlighting that consumer decisions are primarily driven by how product characteristics, particularly sensory ones, shape overall perceptions.

### 6.1. Implications

The findings of this study have several practical and theoretical implications. For producers and marketers, the results highlight the critical role of sensory experiences in driving consumer preferences for artisanal and goat cheese. By offering opportunities for direct tasting, producers can enhance consumer perceptions, build trust, and ultimately increase purchase intent. Sensory evaluations should be integrated with broader marketing strategies, such as storytelling around artisanal production methods, to further enhance the emotional appeal of the product.

Extrinsic attributes, including sustainability claims and geographic origin, remain valuable tools for shaping consumer perceptions. Producers should leverage these attributes to complement sensory experiences, particularly in competitive markets where differentiation is essential. For policymakers, the findings underline the importance of promoting sustainable farming practices and transparent labeling to meet the growing consumer demand for ethically and environmentally responsible products.

From a theoretical perspective, this research reinforces the importance of sensory attributes’ overstated preferences in the purchase intention and their mediating effect through attitude in shaping consumer preferences.

Finally, these results contribute to the literature in three important ways. First, they reaffirm the dominant role of sensory quality in driving food choice. Second, they demonstrate that sustainability labels may trigger expectation–disconfirmation dynamics, potentially undermining their effectiveness. Third, they underscore the value of integrated explicit–implicit research designs for uncovering the cognitive processes of sustainable consumption.

### 6.2. Limitations and Future Studies

This exploratory study has some limitations. The limited sample size and geographic scope may constrain the generalizability of the findings. However, because this study is exploratory rather than confirmatory, its dual-method approach—combining sensory analysis with stated-preference data—allows us to rigorously test, and potentially disprove, the assumption that “sustainable” automatically equates to “better taste”.

Second, this study focused exclusively on goat cheese, which, while providing valuable insights into a specific niche product, may not fully represent other artisanal or dairy products. Additionally, the experimental setting involved blinded guided tastings, which may not entirely replicate real-world purchasing scenarios.

Future research should both broaden and deepen our exploratory findings by expanding participant demographics and geographic scope, incorporating multiple cultural contexts and food categories, and testing in more naturalistic settings. For instance, in-store tasting events and virtual retail simulations—potentially combined with neurophysiological measures—could help determine whether the expectation–disconfirmation dynamics observed in the laboratory hold true in real-world shopping environments. Additionally, it would be valuable to further investigate the nuances of this mediation effect, particularly in relation to different consumer segments and varying levels of product involvement.

## Figures and Tables

**Table 1:** Socio-economic characteristics of the sample (*n* = 100).

Characteristics	%
Gender	
Male	59.00%
Female	41.00%
Education	
Primary and secondary school	11.00%
High school	38.00%
Graduate	12.00%
Post-graduate	39.00%
Income	
Up to EUR 1500	38.00%
Between EUR 1501 and EUR 2499	32.00%
Between EUR 2500 and EUR 4000	18.00%
Over EUR 4000	12.00%
	Mean (SD)
Age	42.55 (17.29)

**Table 2:** Descriptive statistics of items.

Constructs		Items	Mean	St. Dev
**Sensory Attributes**				
Aroma	ARO.1	Cheese has a pleasant smell	3.96	1.12
	ARO.2	The smell of the cheese is inviting	3.92	1.14
	ARO.3	The cheese has a strong or dominant odor	3.61	1.19
Appearance	APP.1	The cheese has an attractive color	4.07	1.12
	APP.2	The cheese has a visually pleasing texture	3.99	1.03
	APP.3	The cheese has an inviting presentation	4.01	1.02
Taste	TAS.1	Cheese has a rich and complex flavor	3.91	1.06
	TAS.2	The cheese has a balanced taste	3.88	1.08
	TAS.3	The cheese has a lingering aftertaste	3.64	1.23
Texture	TEX.1	Cheese has a smooth and creamy texture	3.67	1.08
	TEX.2	The cheese has a firm, chewy texture	3.92	0.98
	TEX.3	The cheese melts easily in the mouth	3.71	1.02
**Credence and Search Attributes**				
It influences my decision to buy goat cheese…				
Extrinsic characteristics	PRICE	…the price	3.76	1.15
	PROM	…the promotions	3.76	1.16
	BRAND.REP	…the brand reputation	3.62	1.20
	RECK.PACK	…the recyclable packaging	3.67	1.27
Quality claims	ORGANIC.CERT	…the organic certification	3.9	1.21
	DOP.CERT	…the PDO certification	4.03	1.12
	MOUNT.CERT	…mountain product certification	3.98	1.22
	ANIM.CERT	…the indication and/or certification animal welfare	4.01	1.22
Label information	MILK.PROV.	…the place of provenance of the milk	4.27	1.02
	PROD.ORIG.	…the place of processing of the product	4.23	1.11
	NUTR.INFO	…the nutritional information on the label	3.89	1.27
	TASTING.TIPS	…the tasting tips	3.88	1.21
Attitude	ATT	I may be interested in purchasing this cheese	3.69	1.33
Intention to buy	ITB	I intend to buy goat cheese	2.06	1.12

**Table 4:** Structural model results.

Hypotheses	Path	Effect	Path Coefficient	*p*-Value	Result
H1a	Sensory characteristics -> Attitude	Direct	0.320 (***)	0.001	Supported
H1b	Sensory characteristics -> ITB	Direct	0.378 (***)	0.001	Supported
H2a	Extrinsic characteristics -> Attitude	Direct	0.322 (**)	0.002	Supported
H2b	Extrinsic characteristics -> ITB	Direct	−0.107	0.351	Not supported
H3a	Quality claims -> Attitude	Direct	0.228 (*)	0.056	Supported
H3b	Quality claims -> ITB	Direct	0.068	0.610	Not supported
H4a	Label information -> Attitude	Direct	−0.132	0.260	Not supported
H4b	Label information -> ITB	Direct	−0.111	0.391	Not supported
H5a	Attitude -> ITB	Direct	0.230 (**)	0.045	Supported
H5b	Sensory characteristics -> Attitude -> ITB	Indirect	0.073 (*)	0.077	Supported
H5c	Extrinsic characteristics -> Attitude -> ITB	Indirect	0.074 (*)	0.084	Supported
H5d	Quality claims -> Attitude -> ITB	Indirect	0.052	0.161	Not supported
H5e	Label information -> Attitude -> ITB	Indirect	−0.030	0.322	Not supported

Note: *, **, and *** denote significance at 10%, 5%, and 1% levels, respectively.

## Data Availability

The data presented in this study are available on request from the corresponding author.

## Appendix A

**Table A1:** Discriminant validity.

	Sensory Characteristics	Extrinsic Characteristics	Quality Claims	Label Information	Attitude	Intention to Buy
Sensory characteristics	1.000					
Extrinsic characteristics	0.129	1.000				
Quality claims	0.135	0.234	1.000			
Label information	0.071	0.242	0.474	1.000		
Attitude	0.235	0.233	0.169	0.073	1.000	
Intention to buy	0.199	0.014	0.030	0.002	0.129	1.000
AVE	0.537	0.562	0.715	0.702	1.000	1.000

**Table A2:** Multicollinearity check (variance inflated factors—VIFs).

	Attitude	Intention to Buy
Sensory characteristics	1.22	1.38
Extrinsic characteristics	1.47	1.63
Attitude		1.59
Label information	2.03	2.06
Quality claims	2.10	2.18
